# A Snapshot of Antimicrobial Resistance in Semi-Wild Oryx: Baseline Data from Qatar

**DOI:** 10.3390/antibiotics14030248

**Published:** 2025-03-01

**Authors:** Asma Mushahidur Rahman, Salma E. Ahmed, Shayma A. Osman, Radhia A. Al-Haddad, Abdallah Almiski, Ristha Kamar, Hana Abdelrahman, Issmat I. Kassem, Andrea Dogliero, Nahla O. Eltai

**Affiliations:** 1 Biomedical Research Centre, Microbiology Department, Qu Health, Qatar University, Doha P.O. Box 2713, Qatar; ar1906263@student.qu.edu.qa (A.M.R.); sa1905660@student.qu.edu.qa (S.E.A.); so1705259@student.qu.edu.qa (S.A.O.); ristha.k@qu.edu.qa (R.K.); hana.abdelrahman@qu.edu.qa (H.A.); 2 International School for Medical Science and Engineering, Doha P.O. Box 7582, Qatar; radhiaghalia@gmail.com (R.A.A.-H.); abdallah.almiski@gmail.com (A.A.); 3 Centre for Food Safety, University of Georgia, Griffin, GA 30609, USA; issmat.kassem@uga.edu; 4 Faculty of Agricultural and Food Sciences, American University of Beirut, Riad El Solh, Beirut 1107, Lebanon; 5 Department of Natural Reserves, Ministry of Environment and Climate Change, Doha P.O. Box 7634, Qatar; andrea.dogliero@gmail.com

**Keywords:** antimicrobial resistance (AMR), *E. coli*, wildlife, Arabian oryx, One Health, ESBL

## Abstract

**Background/Objectives:** The spread of antimicrobial resistance (AMR) is a growing global health concern. Wild animals can play an important role in the amplification and dissemination of AMR and in conservation efforts aiming at controlling diseases in vulnerable wild animal populations. These animals can serve as reservoirs for antibiotic resistance genes and are key in the spread of AMR across ecosystems and hosts. Therefore, monitoring AMR in wild animals is crucial in tackling the spread of resistance in the environment and human population. This study investigated the phenotypic and genotypic resistance of *Escherichia coli* (*E. coli*) isolated from semi-wild oryx (*Oryx leucoryx*) in Qatar. **Methods:** One hundred fecal samples were collected from oryx in diverse natural reserves across Qatar. A selective agar medium was used to isolate *E. coli*, and the identity of the isolates was further confirmed using the VITEK^®^ 2 Compact system. The Kirby–Bauer disk diffusion method was used to test antibiotic susceptibility. Genetic resistance determinants were identified through polymerase chain reaction (PCR) analyses and sequencing using the Oxford Nanopore Technology (ONT). **Results:** The results revealed that 18% (*n* = 18) of the samples harbored *E. coli* with resistance to a single antibiotic, 28% (*n* = 28) were resistant to at least one antibiotic, and 2% (*n* = 2) were multidrug-resistant (MDR). No resistance was observed against colistin. *tetA* and *tetB* encode tetracycline resistance were the most frequently detected genes (57.7%). Whole genome sequencing (WGS) was used to expand on AMR gene-PCR analyses and analyze the resistome of 12*E. coli* isolates. WGS identified several important antibiotic resistance determinates, including *bla_CTX-M_*_-_encoding Extended Spectrum Beta-Lactamase (ESBL) resistance, *soxR* associated with tetracycline target alteration, and *mdtE*, *emrB*, *AcrE*, *mdtF*, and *marA* related to ciprofloxacin efflux pump resistance. **Conclusions:** This study provides essential information regarding AMR in Qatari semi-wild animals, which will guide conservation strategies and wildlife health management in a world experiencing increasing antibiotic-resistant infections. Furthermore, these findings can inform policies to mitigate AMR spread, improve ecosystems, and enhance public and environmental health while paving the way for future research on AMR dynamics in wildlife.

## 1. Introduction

The misuse and abuse of antibiotics have fueled the rise of resistant bacteria among animals and humans, complicating infection treatments and threatening public health [1,2]. Clinically significant antibiotic-resistant bacteria (ARB) are being isolated from various animals, including food animals and wildlife species [3] and synanthropic birds [4,5]. The factors influencing the presence of ARB in wildlife are diverse and remain incompletely understood, with anthropogenic sources, directly or indirectly (e.g., via the environment), playing key roles in the spread of ARB in wildlife [4,6]. Examples of anthropogenic sources include ineffectively treated wastewater draining into natural waters like rivers and lakes and waste from intensive livestock farming [7,8,9,10]. The spread of ARB in the natural environment has harmful economic and health consequences [11]. Therefore, investigations have increasingly focused on wild animals to recognize the existence of antibiotic resistance in diverse environmental sources [12]. If wild animals become colonized with ARB via exposure to contaminated environments, these animals can then amplify ARB and transmit it to pristine environments or agricultural areas [13,14,15]. This will result in ARB amplification and/or transmission cycle across the human, animal, and environment continuum. Therefore, monitoring ARB in wildlife is an integral component of One Health strategies that aim to control the spread of resistance.

*Escherichia coli* (*E. coli*) is a Gram-negative bacterium that resides in the intestinal tract of mammals and birds and has been associated with fecally-contaminated environments [16,17]. Importantly, *E. coli* serves as a paradigm organism for understanding the spread of ARB within specific populations, because monitoring antimicrobial resistance in *E. coli* can serve as an indicator for the emergence and spread of resistance in bacterial communities across hosts and niches [7,18]. Notably, *E. coli* is considered a main element of antimicrobial resistance surveillance programs in food-producing animals. It can act as a reservoir or carrier of antibiotic resistance, further complicating AMR combating efforts. In recent years, antibiotic-resistant (AR) strains of *E. coli* have been detected in livestock farming and wild animals, indicating transmission of resistance outside the clinical and agricultural settings [19]. The detection of AR *E. coli* in wildlife has been associated with inadequately treated human and livestock waste, highlighting the spread of resistance into the broader environment [20]. Therefore, monitoring *E. coli* isolated from wild animals is an effective approach for assessing the spread of AMR bacteria into the environment.

Many studies have demonstrated that the bacteria found in wildlife, including important sequence types and strains carrying clinically relevant antibiotic resistance determinants, were associated with those found in humans and other animals [21]. Subsequently, AMR research in wild animals also helps in deciphering the complex dynamics of resistance dissemination across ecosystems and hosts and via zoonosis and reverse zoonotic pathways. Despite the importance of wildlife and the environment in the various One Health approaches targeting AMR, there is a significant gap in research on AMR in wildlife species, particularly endangered ones. This gap limits our understanding of how health threats (e.g., AMR or infectious agents) in wildlife species affect ecosystems (e.g., transmission of disease across wildlife and other environmental reservoirs), biodiversity (e.g., decline of wildlife due to anthropozoonotic disease), and human health (e.g., zoonosis). Taken together, studying AMR and infectious diseases in wild animals aids biodiversity conservation and expands the One Health approach to include species that can impact the ecosystem and human health.

In this study, we targeted wild animals, because they serve as valuable indicators of environmental and public health [22]. Specifically, we focused on *Oryx leucoryx* (Arabian Oryx), an endangered species native to the desert and steppe regions of the Arabian Peninsula [23]. The Arabian oryx holds significant cultural and national importance in Qatar, where it is recognized as the national animal, symbolizing the country’s heritage and connection to its desert environment. It represents national pride and the ongoing efforts to preserve Qatar’s natural heritage [24]. Classified as endangered and extinct in the wild by the International Union for Conservation of Nature (IUCN) Red List [25] the Arabian oryx has benefitted from several captive breeding and reintroduction programs across the Middle East, in collaboration with international organizations. As a result, the oryx’s status was downgraded to vulnerable in 2011. Studying AMR in endangered or vulnerable species addresses a gap in understanding how AMR might contribute to biodiversity loss, ecosystem degradation and the spread of zoonotic disease. These species inhabit unique ecosystems with under-researched health dynamics, and their conservation helps in preventing broader public health by bridging wildlife conservation and public health under a One Health strategy.

In Qatar, the Arabian oryx is among the four most iconic national animals, alongside the Arabian horse, the dromedary or Arabian camel, and falcons. For this reason, the State of Qatar, particularly the Ministry of Environment and Climate Change (MECC), has been implementing a captive breeding and conservation project for over 16 years to prevent the oryx from becoming extinct. This study fills an important knowledge gap, as there is no data on ARB in Qatar’s wild and semi-wild animals. *Oryx leucoryx* was chosen because of its importance to Qatar’s semi-wild ecosystems, where this species may be exposed to environmental pollutants and AMR pathogens. This research goes beyond conservation, offering new insights into the role of wildlife, particularly endangered or vulnerable species, in AMR transmission, a largely overlooked aspect in the Middle Eastern region. We aim to benchmark the prevalence of antibiotic-resistant *E. coli* strains in Qatar’s semi-wild animals and establish a nationwide monitoring system to track ARB in wildlife. Understanding the extent of AMR in these animal populations is essential for effective surveillance and preventive measures to mitigate the insidious health impacts of AMR on humans, animals, and the environment.

## 2. Results

### 2.1. Demographic Data

In this study, 100 *E. coli* were isolated from otherwise healthy semi-wild oryx, 33% of the isolates (*n* = 33) were from males and 67% (*n* = 67) from females, and all the isolates were screened for AMR. Demographic data included the age and gender of the oryx and the collection locations. Notably, 49% (*n* = 49) of the isolates were collected from young animals (under three years old), while 51% (*n* = 51) were from adults (more than 8 years old). Additionally, 24% (*n* = 24) of the isolates were from the northwestern region, 50% (*n* = 50) from the central zone, and 26% (*n* = 26) from the southwestern part of Qatar. Since this is the first-ever study on AMR in Oryx, it was important to collect demographic data, which would serve two main purposes: (1) identify if age and/or gender influenced AMR carriage in the Oryx, which will help in conservation efforts, and (2) determine if the location of the animal might have contributed to acquisition of AMR. The latter might reveal anthropogenic influencing factors such as pollution of the location.

### 2.2. Detection of Phenotypically Antimicrobial Resistant E. coli

**Figure 1:**
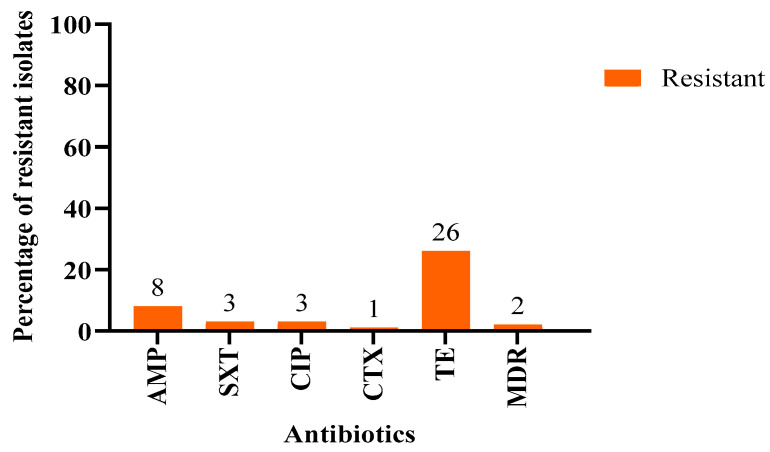
The percentages of resistance to each antibiotic and multidrug resistance (MDR) in *E. coli* isolated from *Oryx leucoryx*. AMP: Ampicillin; CIP: Ciprofloxacin; TE: Tetracycline; SXT: Trimethoprim/Sulfamethoxazole; CTX: Cefotaxime. MDR: multidrug-resistant.

Antimicrobial resistance profiles of the *E. coli* isolates were assessed using 15 antibiotics as detailed in Table 1. Twenty-eight *E. coli* isolates were at least resistant to one of the tested antibiotics (Table A1). Resistance was observed only against five antibiotics: ampicillin, trimethoprim-sulfamethoxazole, ciprofloxacin, cefotaxime, and tetracycline. Among the 100 *E. coli* isolates, 8% exhibited resistance to ampicillin, while 26% of the isolates were resistant to tetracycline. Additionally, 3% of the isolates were resistant to trimethoprim-sulfamethoxazole and ciprofloxacin, while 1% were resistant to cefotaxime and 2% were multidrug- resistant (MDR) as shown in Figure 1. The profiles of the MDR isolates were AMP-SXT-CTX-TE and AMP-SXT-TE, respectively.

**Figure 2:**
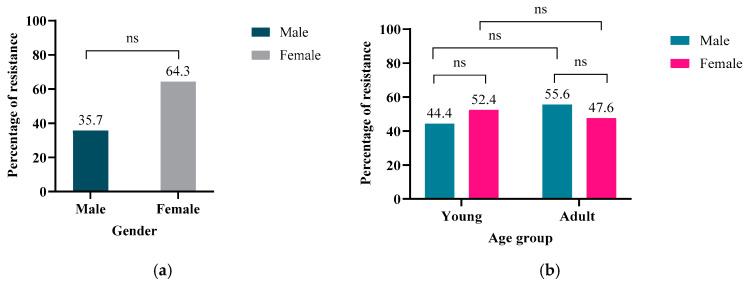
(**a**) The percentages of resistant *E. coli* isolates (out of 28 resistant isolates from a total of 100) among males and females from Qatari semi-wild Oryx leucoryx. Using SPSS statistical software, a higher percentage of resistance was detected among females than males, with 64.3% (*n* = 18/28) and 35.7% (*n* = 10/28), respectively. This difference was not statistically significant (*p* > 0.05). (**b**) Comparing the number of resistant *E. coli* isolates among different age groups and genders. The adult males showed a higher resistance percentage than adult females. ns: not significant.

**Figure 3:**
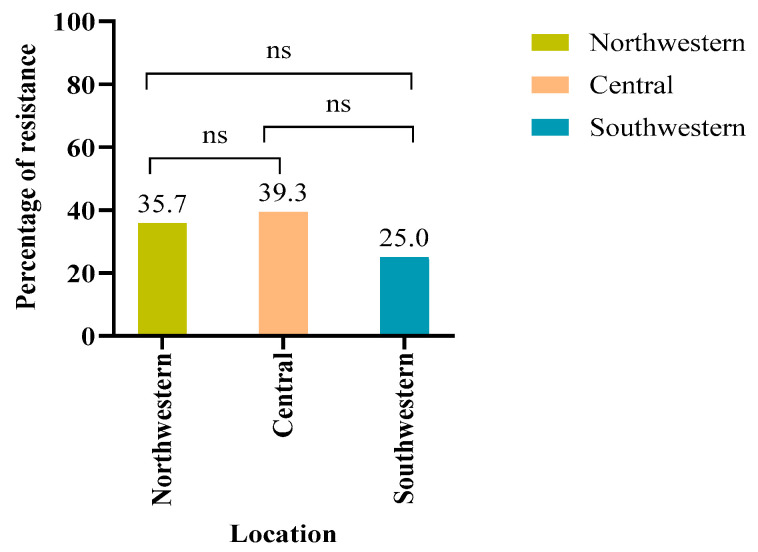
The percentages of resistant *E. coli* isolates across northwestern, central, and southwestern locations. The results did not show statistically significant differences. The highest percentage of resistance was detected in the central region. ns: not significant.

Analysis using SPSS (Version 29.0.0.0) indicated a higher percentage of antibiotic-resistant *E. coli* among female oryx (64.3%, *n* = 18/28) compared to males (35.7%, *n* = 10/28), although this difference was not statistically significant (*p* > 0.05) (Figure 2a). Age-related carriage of resistant isolates was equally distributed, with both young and adult groups yielding resistance in 50% (*n* = 14/28) of the samples (Figure 2b). In the young group, females exhibited higher carriage of resistant *E. coli* (52.4%, *n* = 11/21) compared to males (44.4%, *n* = 8/18) (Figure 2b). Conversely, the adult group showed higher carriage of resistant *E. coli* in males (55.6%, *n* = 10/18) compared to females (47.6%, *n* = 10/21) (Figure 2b). Regarding sample location, the carriage of resistant *E. coli* was highest in the central region (39.3%, *n* = 11), followed by the northwestern (35.7%, *n* = 10) and southwestern (25%, *n* = 7) locations (Figure 3). However, the binary logistic regression analysis (Table A1) yielded a *p*-value > 0.05 for resistance categorized by age, gender, and location, indicating no statistically significant relationship between these factors and the observed resistance percentages.

### 2.3. Screening Genetic Determinants of Resistance Using AMR Gene-Specific PCR Analyses

**Figure 4:**
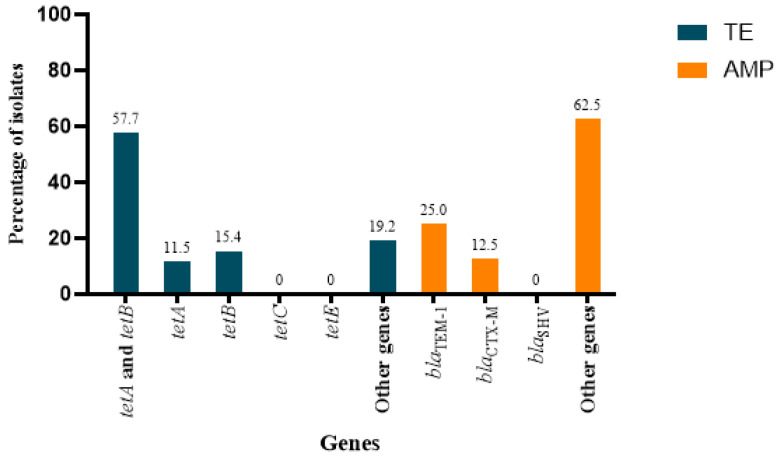
The percentage of genes associated with the phenotypic resistance to 2 antibiotics, tetracycline and ampicillin, was determined using gene-specific PCR. The “Other Genes” were determined using WGS and are listed in Table 2.

PCR analysis revealed that 57.7% of the tetracycline-resistant *E. coli* isolates carried both the *tetA* and *tetB* genes. Additionally, 11.55% of the isolates contained were positive only for *tetA*, while 15.4% were positive only for *tetB*. (Figure 4). *tetC* and *tetE* were not detected by PCR. For the eight ampicillin-resistant isolates, 12.5% (*n* = 1/8) harbored *bla*_CTX-M_, while 25% (*n* = 2/8) of the isolates were positive for *bla*_TEM-1_. The remaining 62.5% (*n* = 5/8) of the ampicillin-resistant isolates did not have any of the four PCR-screened genes associated with ampicillin resistance. None of the ampicillin-resistant *E. coli* tested positive for *bla*_SHV_.

### 2.4. Whole Genome Sequencing Using the Oxford Nanopore Technology (ONT)

Whole Genome Sequencing was performed on 12 *E. coli* isolates that revealed phenotypic resistance against ampicillin, ciprofloxacin, tetracycline, or cefotaxime but their resistance genetic determinants were not detected by PCR. The sequencing results revealed the resistance mechanisms, including relevant mutations and genes, which are listed in Table 2.

## 3. Discussion

The One Health approach has been implemented in Qatar as a multidisciplinary framework to address the increasing public health concern posed by AMR. The National Antimicrobial Resistance Action Plan (2024–2030) of Qatar illustrates the interconnectedness of people, animals, and the environment [26]. By studying AMR in wild animals, this study addresses a knowledge gap that will enhance this action plan and provide insights on antibiotic resistance acquisition and spread in these nationally important hosts. Notably, research on antibiotic resistance is limited in Qatari wild and semi-wild species. Consequently, this study evaluated antibiotic resistance in *E. coli* isolated from the semi-wild Qatari Oryx (*Oryx leucoryx*).

In this study, 28% (*n* = 28) of the bacterial isolates exhibited resistance to at least one antibiotic. The most common resistance was observed to tetracycline in 26% (*n* = 26) of the isolates, followed by ampicillin (8%, *n* = 8), trimethoprim-sulfamethoxazole (3%, *n* = 3), ciprofloxacin (3%, *n* = 3), and cefotaxime (1%, *n* = 1). Additionally, 2% of the isolates were identified as multidrug-resistant (MDR). These findings indicate a relatively low resistance level compared to similar studies in other regions. For instance, in a study conducted in Costa Rica, 93% (*n* = 63) of the bacterial isolates showed resistance to cephalexin (58%, *n* = 39), ampicillin (43%, *n* = 29), and oxytetracycline (22%, *n* = 15). The bacterial isolates in that study were sourced from *Bradypus variegatus* (Three-Toed Sloth), *Choloepus hoffmanni* (Two-Toed Sloth), and *Alouatta palliata* (Howler Monkey). Notably, 48% (*n* = 32) of the isolates in the Costa Rican study were classified as MDR, highlighting a significantly higher prevalence of resistance compared to our findings [27]. The differences in antibiotic resistance observed between the two studies are likely attributable to variations in the targeted animal populations, geographic locations, antibiotic practices and sampling techniques among others. In the Costa Rican study, the samples were collected from wild animal rehabilitation facilities, where the isolates were divided into two groups: 30% from restored animals and 70% from recently admitted animals. Remarkably, 21% of the rehabilitated animals had received prior antibiotic treatment, which may have contributed to the higher resistance rates observed in that study [27]. Antibiotics are infrequently used in healthy, semi-wild *Oryx leucoryx,* and are primarily reserved for treating diseased animals in Qatar. The main antibiotics employed in these cases include tetracyclines (e.g., oxytetracycline), fluoroquinolones (e.g., marbofloxacin, enrofloxacin), and beta-lactams (e.g., amoxicillin) (personal communication). Therefore, the relatively low levels of resistance observed in our study, particularly to ampicillin, ciprofloxacin, and tetracycline, may be attributed to the limited and targeted use of antibiotics. In contrast, a study by Alhababi et al. (2020) [28] investigating AMR profiles in food animals in Qatar, specifically cattle, camels, and pigeons, found a 42.8% resistance rate (*n* = 114/266). Furthermore, resistance rates were higher at 36.5% in cattle, 20.6% in camels, 70% in pigeons, and 90% in broiler chickens. In comparison, the restricted and focused use of antibiotics in Oryx probably explains the reduced resistance rates seen in this study [28]. This highlights the significant differences in resistance patterns between wild and domesticated animal populations, potentially due to variations in antibiotic use and exposure [28]. The oryx in this study inhabited a semi-conserved area, which is separated from livestock and wastewater sources. As a result, the oryx should have no direct contact with these potential environmental contributors to AMR. While separation likely plays a role in the relatively low levels of AMR observed within this population, other contributing factors such as dust storms can potentially transport AMR from distant sources to the regions occupied by the oryx [29].

Alhababi et al. [28] also reported that the highest resistance in *E. coli* isolated from food animals in Qatar was observed against tetracycline (64% in pigeons, 27.9% in cattle, and 15% in camels), followed by ampicillin (55.1%, 14%, and 7%, respectively) [28]. In our study, comparatively modest resistance rates to ampicillin (8%) and tetracycline (26%) were also identified in the oryx. The widespread use of tetracycline and ampicillin globally likely contributes to these trends, as ampicillin and tetracycline are commonly used as first-line treatments for animal infections worldwide [30]. This suggests that frequent and widespread antibiotic exposure in food animals might potentially be a significant factor driving the higher resistance rates observed in domesticated species compared to wild animals [31]. Perhaps, it can also be highlighted that the AMR observed in food animals might be difficult to spread to the oryx or their environment in Qatar, likely due to the separation between these animals and proper handling of food animal waste. However, in our study, one multi-drug-resistant *E. coli* isolated from Oryx had an MARI > 0.2, suggesting that it might have originated in high-risk contaminated sources that have experienced frequent use of antibiotics. This latter was concerning and highlighted the need for further investigations into oryx and other wild animals and other factors such as AMR transported by dust to confirm our observations and detect potential sources of AMR that might affect these animals.

This study found a unique profile in one *E. coli* isolate which was resistant to cefotaxime but not ceftazidime, despite both being third-generation cephalosporins. This differential resistance, although not common, can be attributed to specific bacterial mechanisms that selectively affect resistance to these antibiotics. WGS analysis revealed that cefotaxime resistance might be primarily due to efflux pump action and/or antibiotic target changes, not beta-lactamase production (Table 2). The active expulsion by efflux pumps reduces cefotaxime’s intracellular concentration, rendering it ineffective. At the same time, the altered target site may prevent the binding of the antibiotic. Ceftazidime, in our isolate, appears less affected by these resistance mechanisms, possibly due to differences in pumping ability or modified target affinity. The absence of beta-lactamase production in this isolate suggests other mechanisms, possibly involving chromosomal mutations that enhance the bacterium’s resistance to cefotaxime without affecting ceftazidime susceptibility. These findings highlight the importance of comprehensive resistance profiling, including WGS, to identify the mechanisms behind resistance in wildlife samples. Further investigation into the genetic basis of the efflux pumps and target alterations may provide valuable insights into how bacteria adapt to third-generation cephalosporins and how resistance can be differentially expressed between closely related antibiotics.

Taken together, our findings provide valuable insights into AMR in the Oryx, an ecologically important semi-wild species. Although the observed AMR levels were relatively low, this study empahsized the need for continuous monitoring and highlighted the potential impact of AMR on wildlife conservation practices. The data can inform future AMR management strategies by emphasizing the need for targeted interventions in areas with higher AMR risks, including in wildlife populations. Additionally, the study underscores the significance of maintaining isolated environments, like the semi-conserved areas studied here, to minimize wildlife exposure to potential AMR anthropogenic sources. This research could aid in developing AMR management practices within wildlife conservation programs, promoting sustainable and healthy ecosystems.

## 4. Materials and Methods

### 4.1. Sample Collection

**Figure 5:**
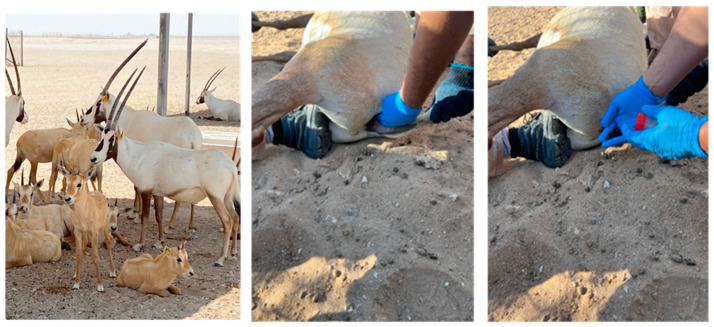
A full view of the studied Arabian Oryx along with two images depicting the sample collection process in this study.

A total of 100 rectal faecal samples were collected from 100 individual *Oryx leucoryx*. Given that there are about 1000 Arabian Oryx in semi-wild conditions currently in Qatar, sampling 100 individual oryx will result in a statistical power of 0.8 for this study. Therefore, the sample number was selected based on statistical guidelines that will provide an adequate representation of the population, capturing key insights while balancing the available resources for AMR analyses from healthy semi-wild Arabian Oryx (*Oryx leucoryx*) between 18 February and 20 May 2024. Along with the samples, information about the age, sex, and antibiotic exposure was also collected. The samples were collected by a qualified veterinarian using sterile techniques (Figure 5) in collaboration with the Ministry of Environment and Climate Change (MECC), Department of Wildlife Protection, from Qatar’s central, northwestern, and southwestern regions. During sample collection, rigorous welfare protocols were implemented to ensure the animals’ well-being throughout the study. Sampling was performed by a trained and experienced veterinarian and technologist following established guidelines designed to minimize stress and discomfort. The animals were approached gently and calmly to reduce anxiety. Additionally, the sampling process was completed swiftly, within approximately 3 min, and was conducted exclusively during the early morning hours, when temperatures are cooler, to avoid any potential thermal shock.

The samples were labelled and transported in cooled boxes (4–8 °C) to the Microbiology Laboratory at the Biomedical Research Center (BRC), Qatar University (QU). Upon arrival, the samples were stored at 4 °C and processed within 24 h. Ethical approval for this study was obtained from the Institutional Biosafety Committee (IBC) at Qatar University, with approval number QU-IBC-005/2024.

### 4.2. Escherichia coli Isolation and Identification

One gram of the faecal sample was suspended in 3 mL of Phosphate-Buffered Saline (PBS, Aton Scientific, Hyde, UK) and vortexed vigorously. Subsequently, 10 μL of each suspension was streaked onto selective CHROMagar^TM^ *E. coli* plates (Hi-Media, Mumbai, India) and incubated at 37 °C for 18 to 24 h [32]. Typical *E. coli* colonies, characterized by a green colour with a smooth surface, were selected and streaked onto nutrient agar plates (Hi-Media, Mumbai, India). The identity of the colonies was confirmed using VITEK^®^ 2 Compact system (bioMerieux, Marcy l’Etoile, France). The confirmed isolates were transferred to Cryovial tubes (Technical Service Consultant, Lancashire, UK) and stored at −80 °C until further analysis.

### 4.3. Phenotypic Antibiotic Susceptibility Testing (AST)

*E. coli* isolates were screened for resistance to 15 important antibiotics (Table 3) (Liofilchem, Roseto degli Abruzzi, Italy) using the standard Kirby–Bauer disk diffusion method as outlined by CLSI (2020) [33]. The microdilution method was used to assess colistin resistance with the SensiTest colistin kit (Liofilchem, Roseto degli Abruzzi, Italy) following the manufacturer’s guidelines.

For the disk diffusion assay, overnight cultures of *E. coli* were suspended in PBS (Aton Scientific, Hyde, UK) to achieve an inoculum equivalent to 0.5 McFarland standard as measured by DensiCHEK™ Plus instrument (bioMéerieux, Craponne, France). Suspensions were spread using swabs on Cation-adjusted Mueller–Hinton agar (MHA) plates (Himedia-India, Maharashtra, India). Then, the antibiotic disks (Liofilchem, Roseto degli Abruzzi, Italy) were applied on the surface of the plate, which was incubated at 37 °C for 18 to 24 h. The zone of inhibition was measured and interpreted according to the CLSI (2020) guidelines [33]. *E. coli* ATCC 25922 and ATCC 35218, a beta-lactamase-producing strain, were used as quality controls (QCs). If any isolate exhibited resistance to at least one agent in three or more antimicrobial categories, it was classified as MDR.

The multiple antibiotic resistance index (MARI) for each resistant isolate was calculated as described in Woh et al. (2023) [34]. MARI > 0.2 suggests that an isolate is from high-risk contaminated sources (that experience frequent use of antibiotics), while MARI ≤ 0.2 suggest that the isolates belong to sources with relatively low antibiotic use (Table A1).

Molecular Detection of Antibiotic Resistance Determinants The genomic DNA of *E. coli* isolates exhibiting phenotypic resistance was extracted using the QIAamp UCP Pathogen Mini Kit (Qiagen, Hilden, Germany) following the manufacturer’s instructions. The concentration and purity of the extracted DNA were assessed using a Nanodrop Lite Spectrophotometer (Thermo Scientific, Waltham, MA, USA). The extracted DNA was then stored at −20 °C until it was needed for further experiments.

The extracted DNA was used to run PCR for the following genes: *tetA, tetB, tetC, tetE, bla*_TEM-1_, *bla*_CTX-M_, *bla*_SHV_ using previously published primers (Table A2). The PCR mixture was made in a volume of 25 μL, using HotStar Taq^®^ Plus Master mix (Qiagen, Hilden, Germany) containing 10 μL of HotStar, 0.5 μL of forward and reverse primer, 9 μL of nuclease-free H_2_O and 2 μL of Red Color reagent as a loading dye, and 3 μL of DNA. The reaction mixture was amplified using a Biometra TAdvanced PCR Thermal Cycler (Analytik Jena, Jena, Germany). Each PCR reaction had specific conditions used for the amplification of specific genes, which are provided in Table A3. *E. coli* NCTC^®^ 13,461™, *E. coli* NCTC ^®^ 13,351™, and *Klebsiella pneumoniae* NCTC ^®^13,368™ were used as positive controls for *bla_CTX_*_-M_ G1, *bla*_TEM_ and *bla* _SHV_, respectively. For *tet* genes, *E. coli* strains isolated from hospital patients and identified as tetracycline-resistant by sequencing were used as controls. The amplified PCR products were subjected to electrophoresis in 1.5% agarose (Agarose-LE, Ambion^®^, Thermo Scientific, Waltham, MA, USA), stained with ethidium bromide (Promega, Madison, WI, USA) for 45 min, and visualized using iBrightTM CL1000 Imaging System (Thermo Fisher, Waltham, MA, USA).

### 4.4. WGS Using Oxford Nanopore Technology (ONT)

WGS analyses targeted only 12 isolates that exhibited phenotypic antibiotic resistance (including one cefotaxime-resistant isolate) but were negative for specific AMR genes by the PCR-based screening. The WGS was performed using the Oxford Nanopore Technology (ONT) protocols (https:// nanoporetech.com/document/ligation-sequencing-amplicons-native-barcoding-v14-sqk-nbd114-24 (accessed on 18 February 2025)).

Briefly, high molecular weight genomic DNA (gDNA) from the isolates was first quantified using a Qubit fluorometer (Thermo Fisher Scientific, Waltham, MA, USA). The DNA was then repaired and prepared for adapter ligation using the NEBNext FFPE Repair Mix (NEB, M6630) and Ultra II End Repair/dA-tailing Module (NEB, E7546) to address nicks, gaps, or deaminated bases, which are critical for preserving DNA. Native barcoding was performed by ligating barcodes (NB01-24) from the Native Barcoding Kit 24 V14 (SQK-NBD114.24) to the DNA ends using NEB Blunt/TA Ligase Master Mix (NEB, M0367), which was followed by addition of EDTA to terminate ligation. Barcoded samples were pooled and excess adapters were removed using 0.4X AMPure XP bead. Sequencing adapters were then ligated to the pooled barcoded DNA using the Quick Ligation Module (NEB, E6056) with a final clean-up using Long Fragment Buffer (LFB) to retain fragments > 3 kb. The R10.4.1 flow cell (FLO-MIN114) was primed to stabilize the nanopore array. A priming mix containing Flow Cell Flush (FCF), Flow Cell Tether (FCT) and Bovine Serum Albumin (BSA; 0.2 mg/mL final concentration) was prepared to minimize pore blockage and enhance DNA capture. After removing the storage buffer and ensuring that there were no air bubbles, the priming mix was loaded into the priming port, followed by a 5 min incubation. The *E. coli* library was diluted in Sequencing Buffer (SB) and Library Beads (LIB) and a total of 75 µL of the library-bead mix was added dropwise to the SpotON sample port to ensure uniform distribution. The flow cell was shielded with a light protector to prevent signal interference. Sequencing was initiated using the MinKNOW software (version 23.11.7) on the GridION 5X GXB01496 platform (Oxford Nanopore Technologies, Oxford Science Park, Oxford, UK). MinKNOW controlled data acquisition, real-time base-calling, and sequencing monitoring performance. The base-called data were demultiplexed to separate individual barcoded samples, and further downstream analysis was performed in a Linux environment. Post-base-calling, quality control was performed using FastQC (version 0.12.1), a bioinformatics tool that generates per-base sequence quality scores, GC content distribution, sequence length distribution, and the presence of overrepresented sequences such as adapters or contaminants. A specialized tool, Porechop, was used to trim adapter sequences and handle chimeric reads by splitting them at the junctions. Furthermore, de novo assembly of the sequence was performed using Flye. The assembly process involved multiple stages, including error correction, repeat graph construction, contig generation, and final polishing. The final assemblies (*n* = 12) are available on the NCBI website under BioProject (the genome accessions are listed in Table 2). The assembled sequences were analyzed using CARD: RGI analyzer software (https://card.mcmaster.ca/analyze/rgi, accessed on 18 February 2025) to identify the AMR genes and determinants responsible for resistance [35].

### 4.5. Data Analysis

Data were imported into Microsoft Excel 2016 to generate figures and run analyses to compare antibiotic resistance percentages. All the graphs were then generated using GraphPad Prism (Version 10.4.0). The demographic data, including the age and gender and the location of the oryx, were statistically analyzed using the Statistical Package for Social Sciences (SPSS) (IBM SPSS Statistics, Version 29.0.0.0). Oryx under 3 years of age were considered young, and 3 to 8 years old were categorized as adults. A binary logistic regression test was conducted to examine the correlation and statistical significance between age, gender, and location for the observed antibiotic resistance. The analysis used a confidence interval of 95%. *p*-value < 0.05 was considered statistically significant, while a *p*-value > 0.05 was deemed statistically insignificant.

## 5. Conclusions

This study establishes a crucial preliminary baseline for AMR in oryx, highlighting the current low resistance levels. Early AMR trend identification allows for proactive measures to prevent future increases in resistance. The findings will inform policy decisions and guide Oryx antibiotic stewardship programs, helping the country maintain low AMR levels in these national animals. Overall, the study supports sustainable practices to protect wildlife and informs future management strategies to disrupt the AMR cycle that affects humans, animals, and the environment. Future research should build on the findings of this study by tracking AMR trends in oryx over time and expanding the scope to include additional bacterial species and antimicrobial agents. It is also essential to examine environmental factors that may contribute to resistance (such as dust storms) and to conduct comparative studies with other wildlife species to identify common risk factors. Furthermore, investigating the potential for zoonotic and anthropozoonosis transmission of AMR (e.g., between oryx and human caretakers, and conservationists) will provide valuable insights.

The study has some limitations, including focusing mainly on *E. coli*, and potentially overlooking other relevant resistance patterns. Furthermore, selecting *E. coli* on agar without using specific antibiotics might have favored susceptible strains. Other relevant environmental factors were not extensively evaluated, which could provide important insights linking the oryx habitats to human-affected sources. Additionally, the study offers cross-sectional data without tracking long-term trends. Lastly, the oryx ecology should have been analyzed further to identify further exposure to AMR sources.

## Figures and Tables

**Table 1:** Resistance of Escherichia coli isolated from Oryx to individual antibiotics.

No.	Antibiotic	Concentration	Susceptible (n, %)	Resistant (n, %)
1	Ampicillin (AMP)	10 μg	92 (92%)	8 (8%)
2	Amoxicillin-clavulanic acid (AUG)	30 μg	100 (100%)	0 (0%)
3	Piperacillin-tazobactam (TZP)	25 μg	100 (100%)	0 (0%)
4	Ertapenem (ETP)	10 μg	100 (100%)	0 (0%)
5	Meropenem (MRP)	10 μg	100 (100%)	0 (0%)
6	Amikacin (AK)	30 μg	100 (100%)	0 (0%)
7	Gentamicin (CN)	10 μg	100 (100%)	0 (0%)
8	Fosfomycin (FOS)	200 μg	100 (100%)	0 (0%)
9	Trimethoprim-sulfamethoxazole (SXT)	25 μg	97 (3%)	3 (3%)
10	Ciprofloxacin (CIP)	5 μg	97 (3%)	3 (3%)
11	Cefotaxime (CTX)	30 μg	99 (99%)	1 (1%)
12	Ceftazidime (CAZ)	30 μg	100 (100%)	0 (0%)
13	Nitrofurantoin (F)	300 μg	100 (100%)	0 (0%)
14	Tetracycline (TE)	30 μg	74 (74%)	26 (26%)
15	Colistin (Broth microdilution)	0.25–16 mg/mL	100 (100%)	0 (0%)

**Table 2:** Phenotypic resistant profiles of *E. coli* isolates identifying AMR genes using ONT genome sequencing technique.

Isolate No.	Phenotypic Resistance	AMR Genes	Drug Class	Resistance Mechanism	Accession Number	Bio Project Number
91	Tetracycline	*acrS, kpnE*	Tetracycline	Antibiotic efflux	JBKFEU000000000	PRJNA1203416
11	Tetracycline	*soxR* with mutation	Tetracycline	Antibiotic target alteration Antibiotic efflux	JBKFDZ000000000	PRJNA1203247
34	Ciprofloxacin	*emrA*	Fluoroquinolone antibiotic	Antibiotic efflux	JBKFEA000000000	PRJNA1203252
32	Ampicillin	*acrAB-toIC* with *acrR* mutation	Penicillins	Antibiotic target alteration Antibiotic efflux	JBKFEO000000000	PRJNA1203389
29	Tetracycline, ampicillin	*kpnE*	Tetracycline	Antibiotic efflux		
		*fabl* mutations	Multi-drug	Antibiotic target alteration	JBKFEP000000000	PRJNA1203404
35	Tetracycline	*kpnE*	Tetracycline	Antibiotic efflux	JBKFEQ000000000	PRJNA1203405
47	Ampicillin	*qacG*	Small MDR antibiotic efflux pump	Antibiotic efflux	JBKFES000000000	PRJNA1203414
55	Ampicillin	*acrAB-toIC* with *marR* mutations	Multi-drug	Antibiotic target alteration Antibiotic efflux	JBKFET000000000	PRJNA1203415
92	Ampicillin	*acrAB-toIC* with *acrR* mutation	penicillin	Antibiotic target alteration Antibiotic efflux	JBKFEV000000000	PRJNA1203417
39	Cefotaxime	*soxR* with mutation	Cephalosporin drug	antibiotic target alteration, antibiotic efflux	JBKFER000000000	PRJNA1203408
78	Ciprofloxacin	*mdtE, emrB, AcrE,mdtF* and *marA*	Fluoroquinolone	Antibiotic efflux	CP182564	PRJNA1224957
06	Ciprofloxacin	*mdtE, emrB, AcrE,* *mdtF and marA*	Fluoroquinolone	Antibiotic efflux	CP180189	PRJNA1218299

**Table 3:** List of antibiotics used in the experiments with corresponding categories, concentrations, and CLSI 2020 susceptibility range (mm).

No.	Antibiotic	Antibiotic Class	Concentration	CLSI Susceptibility Range (mm)
1	Ampicillin (AMP)	Penicillin	10 μg	≥17 S/R 13≤
2	Amoxicillin-clavulanic acid (AUG)	Penicillin	30 μg	≥18 S/R 13≤
3	Piperacillin-tazobactam (TZP)	Penicillin-beta-lactamase inhibitor	25 μg	≥21 S/R 17≤
4	Ertapenem (ETP)	Carbapenem	10 μg	≥22 S/R 18≤
5	Meropenem (MRP)	Carbapenem	10 μg	≥23 S/R 19≤
6	Amikacin (AK)	Aminoglycoside	30 μg	≥17 S/R 16≤
7	Gentamicin (CN)	Aminoglycoside	10 μg	≥15 S/R 12≤
8	Fosfomycin (FOS)	phosphonic acid derivative	200 μg	≥16 S/R 12≤
9	Trimethoprim-sulfamethoxazole (SXT)-Sulfonamide,		25 μg	≥ 16 S/R 10 ≤
10	Ciprofloxacin (CIP)	Fluoroquinolone	5 μg	≥21 S/R 15≤
11	Cefotaxime (CTX)	Cephalosporin	30 μg	≥26 S/R 22≤
12	Ceftazidime (CAZ)	Cephalosporin	30 μg	≥21 S/R 17≤
13	Nitrofurantoin (F)	Nitrofuran	300 μg	≥17 S/R 14≤
14	Tetracycline (TE)	Tetracycline	30 μg	≥15 S/R 11≤
15	Colistin (Broth microdilution)	Polymyxin	0.25–16 mg/mL	≤1 S/R 4≥

## Data Availability

The genome assemblies included in this study are available at the NCBI website (https://www.ncbi.nlm.nih.gov/bioproject, accessed on 1 January 2025) The BioProject number and assembly accessions are listed in Table 2.

## Appendix A

**Table A1:** Phenotypic resistant profiles of *E. coli* isolates from Oryx leucoryx rectal swabs (*n* = 100).

Resistance Phenotype	Frequency	Percentage (%)	Multiple Antimicrobial Resistance Indices (MARI)
CIP; TE	3	3	0.14
AMP; TE	4	4	0.14
* AMP; SXT; CTX; TE	1	1	0.26
AMP; SXT	1	1	0.14
* AMP; SXT; TE	1	1	0.2
No resistance	72	72	Not applicable
Resistant to only one antibiotic	18	18	Not applicable

* MDR multidrug-resistant; AMP: Ampicillin; CIP: Ciprofloxacin; TE: Tetracycline; SXT: Trimethoprim/Sulfamethoxazole; CTX: Cefotaxime. MARI was calculated as described in Woh et al. (2023) [34]. MARI > 0.2 suggests that an isolate is from high-risk contaminated sources (that experience frequent use of antibiotics), while MARI ≤ 0.2 suggests that the isolates belong to sources with relatively low antibiotic use.

**Table A2:** Primer sequences for *tetA*, *tetB*, *tetC*, *tetE*, *bla*_TEM-1,_ *bla*_CTX-M_, *bla*_SHV_ targeted by PCR and the amplified fragment size.

Antibiotic	Target Gene	Primer Sequence (5′–3′)	PCR Fragment Size (bp)	References
Tetracycline (TE)	*tetA*	F: GCT ACA TCC TGC TTG CCT TC	210	[36]
		R: CAT AGA TCG CCG TGA AGA GG		
	*tetB*	F: TTG GTT AGG GGC AAG TTT TG	659	[36]
		R: GTA ATG GGC CAA TAA CAC CG		
	*tetC*	F: CTT GAG AGC CTT CAA CCC AG	418	[37]
		R: ATG GTC GTC ATC TAC CTG CC		
	*tetE*	F: AAA CCA CAT CCT CCA TAC GC	278	[37]
		R: AAA TAG GCC ACA ACC GTC AG		
Ampicillin (AMP)	*bla*_TEM-1_	F: TCG CCG CAT ACA CTA TTC TCA GAA TGA	431	[38]
		R: CTG ACT CCC CGT CGT GTA GAT A		
	*bla*_CTX-M_	F: ACG CTC ACC GGC TCC AGA TTT AT	688	[39]
		R: CGATATCGTTGGTGGTGCCATA		
	*bla*_SHV_	F: GGG TTA TTC TTA TTT GTC GCT	567	[38]
		R: TTAGCGTTGCCAAGTGCTC		

**Table A3:** PCR conditions for *tetA*, *tetB*, *tetC*, *tetE*, *bla*_TEM-1,_ *bla*_CTX-M_, and *bla*_SHV_ targeted genes.

Target Gene	PCR Conditions (Temperature/Duration)				
	Initial Denaturation	Denaturation	Annealing	Extension	Final Extension
*tetA*	95 °C for 5 min	94 °C for 1 min	55 °C for 1 min	72 °C for 1 min (35 cycles)	72 °C for 5 min
*tetB*	95 °C for 5 min	94 °C for 1 min	55 °C for 1 min	72 °C for 1 min (35 cycles)	72 °C for 5 min
*tetC*	95 °C for 5 min	94 °C for 1 min	55 °C for 1 min	72 °C for 1 min (35 cycles)	72 °C for 5 min
*tetE*	95 °C for 5 min	94 °C for 1 min	55 °C for 1 min	72 °C for 1 min (35 cycles)	72 °C for 5 min
*bla*_TEM-1_	96 °C for 5 min	96 °C for 30 s	44 °C for 45 s	72 °C for 60 s (35 cycles)	72 °C for 10 min
*bla*_CTX-M_	95 °C for 5 min	94 °C for 30 s	60 °C for 40 s	72 °C for 1 min (30 cycles)	72 °C for 7 min
*bla*_SHV_	95 °C for 5 min	94 °C for 1 min	58 °C for 1 min	72 °C for 1 min (35 cycles)	72 °C for 5 min
